# A Multimodal Large Language Model Framework for Intelligent Perception and Decision-Making in Smart Manufacturing

**DOI:** 10.3390/s25103072

**Published:** 2025-05-13

**Authors:** Tianyu Wang, Bowen Zhang, Daqi Jiang, Dong Li

**Affiliations:** 1 State Key Laboratory of Robotics, Shenyang Institute of Automation, Chinese Academy of Sciences, Shenyang 110016, China; wangtianyu@sia.cn; 2 Shenyang Institute of Automation, Chinese Academy of Sciences, Shenyang 110016, China; 3 National Frontiers Science Center for Industrial Intelligence and Systems Optimization, Northeastern University, Shenyang 110819, China; jiangdaqi@mail.neu.edu.cn

**Keywords:** multimodal large language model, smart manufacturing, semantic tokenization, Transformer model, decision-making

## Abstract

In modern manufacturing, making accurate and timely decisions requires the ability to effectively handle multiple types of data. This paper presents a multimodal system designed specifically for smart manufacturing applications. The system combines various data sources including images, sensor data, and production records, using advanced multimodal large language models. This approach addresses common limitations of traditional single-modal methods, such as isolated data analysis and poor integration between different data types. Key contributions include a unified method for representing different data types, dynamic semantic tokenization for better data processing, strong alignment strategies across modalities, and a practical two-stage training method involving initial large-scale pretraining and later fine-tuning for specific tasks. Additionally, a novel Transformer-based model is introduced for generating both images and text, significantly improving real-time decision-making capabilities. Experiments on relevant industrial datasets show that this method consistently performs better than current state-of-the-art approaches in tasks like image–text retrieval and visual question answering. The results demonstrate the effectiveness and versatility of the proposed methods, offering important insights and practical solutions to enhance intelligent manufacturing, predictive maintenance, and anomaly detection, thus supporting the development of more efficient and reliable industrial systems.

## 1. Introduction

In modern industrial settings, production tasks are growing increasingly complex, driving an urgent demand for advanced intelligent systems capable of multimodal data perception, analysis, and fusion-based decision-making [1,2]. These systems must be designed to manage the collaborative processing of multi-source heterogeneous data, including visual information, sensor signals, and production records. Such data are inherently characterized by pronounced temporal dependencies and heterogeneity, posing significant challenges for conventional single-modal processing methods. These methods often fall short of capturing the latent relationships between modalities, thereby limiting the effectiveness of intelligent perception systems in real-world applications. As a result, issues such as data silos and undesirable decision-making accuracy often arise, thus preventing the overall efficiency and responsiveness of industrial operations.

Achieving robust multimodal data fusion, however, remains a formidable challenge. The diverse modalities involved exhibit significant disparities in structure, feature space, and temporal resolution. For example, image data typically embody high-dimensional static representations; time-series data are expressed as low-dimensional dynamic sequences; and text data consist of highly semantic, unstructured content. The interactions among these modalities are often nonlinear and intricately complex. Traditional industrial intelligent systems tend to process each modality in isolation, resulting in fragmented analysis workflows that are inefficient and incapable of uncovering deeper cross-modal associations. In this context, multimodal data fusion emerges as a critical enabling technology. By integrating visual, temporal, and textual information into a unified feature representation space, a more holistic and nuanced understanding of industrial system complexities can be attained. The ability to perform such fusion and collaborative analysis is fundamental to propelling industrial automation toward higher levels of intelligence, adaptability, and sophistication. Large multimodal models have demonstrated remarkable potential in this regard, offering robust mechanisms for the efficient fusion of heterogeneous data types such as images, sensor readings, and production logs. Compared to traditional single-modal models, these architectures exhibit superior generalization capabilities and enhanced robustness, making them well suited to the demands of real-time perception and intelligent decision-making in dynamic and complex industrial environments. Their core strength lies in the capacity to encode disparate modalities within a shared feature space, enabling deep semantic understanding and intelligent inference across data types. This capability is pivotal for achieving high-precision perception and automated decision support in modern industrial contexts.

Recent advancements in models such as GPT-4o, LLaMA, and DeepSeek have demonstrated transformative progress in multimodal data understanding and generation tasks, driven by large-scale pretraining and sophisticated cross-modal alignment strategies [3,4,5,6]. Extending the fusion mechanisms of these large multimodal models to the domain of industrial multi-source data analysis holds tremendous promise. It can effectively dismantle data silos, unlock hidden cross-modal relationships, and provide robust theoretical and technological foundations for real-time perception, predictive analysis, and decision-making in industrial applications.

**Figure 1:**
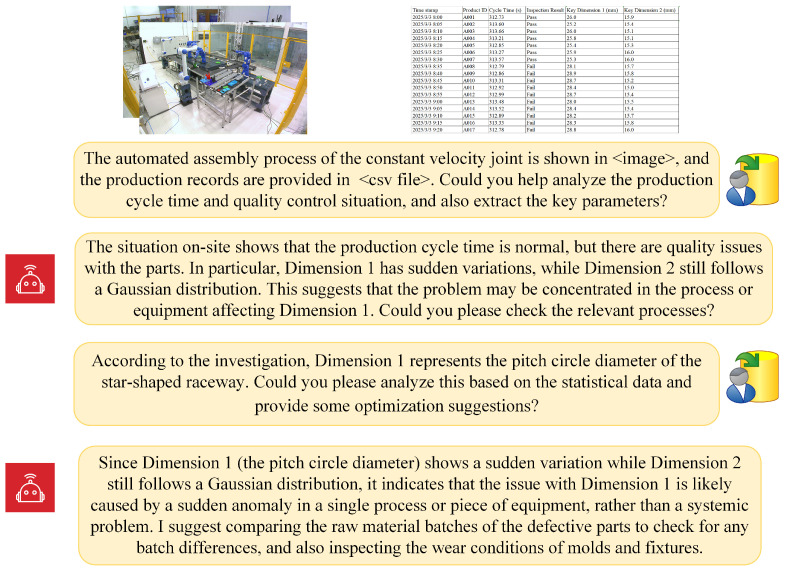
Dialogue example of a multimodal large language model-based Q&A system for smart manufacturing.

Building on this foundation, the present study proposes the development of a multimodal intelligent perception and decision-making system tailored for complex industrial scenarios. By harnessing the complementary characteristics of image data, time-series signals, and unstructured text, we introduce a unified multimodal representation learning framework. This framework employs a two-stage strategy of large-scale pretraining followed by task-specific fine-tuning, enabling the model to effectively adapt to diverse industrial applications. The core research contributions encompass the design of robust multimodal data alignment methodologies, the formulation of scalable pretraining strategies, the implementation of few-shot fine-tuning techniques for industrial deployment, and the construction of a unified bidirectional image–text generation model [7,8]. Collectively, these advancements culminate in the realization of an industrial question answering and decision-making system, illustrated in Figure 1. This system is capable of delivering accurate, efficient, and real-time intelligent Q&A services to support critical industrial functions such as production management, equipment maintenance, fault diagnosis, and anomaly detection, thereby markedly enhancing decision-making efficiency and elevating the overall intelligence of production environments.

In conclusion, the development of multimodal intelligent perception and decision-making systems has emerged as a pivotal requirement in the pursuit of next-generation intelligent manufacturing and industrial automation [9,10]. Despite substantial progress, current approaches continue to face formidable challenges in achieving deep, meaningful fusion of multimodal data and supporting high-quality, real-time decision-making in industrial contexts. This study aims to overcome these limitations by constructing a novel industrial intelligent perception and decision-making framework powered by large-scale multimodal models [11]. The proposed framework emphasizes cross-modal feature alignment and unified semantic modeling, taking into full consideration the heterogeneous nature of industrial data sources and the complex, dynamic requirements of industrial decision processes.

By uncovering the fundamental mechanisms through which multimodal data fusion can enhance industrial intelligence and decision-making efficacy, this research advances the development of an efficient, scalable, and adaptive optimization and decision-making architecture. The outcomes of this work are poised to deliver critical technological support for key domains including intelligent manufacturing, predictive maintenance, and fault diagnostics. Beyond these applications, the findings will also provide valuable theoretical guidance and practical reference for extending artificial intelligence technologies to increasingly complex industrial environments, paving the way for more autonomous, resilient, and intelligent industrial systems.

## 2. Related Works

### 2.1. Current Progress in Modality Alignment and Instruction Tuning for Multimodal Large Language Models

Large language models (LLMs) have garnered widespread recognition for their transformative impact on natural language processing (NLP) and text generation. Nevertheless, early iterations of these models were predominantly confined to single-modal inputs, presenting substantial limitations when addressing complex tasks that require the integration and analysis of multidimensional signals [12,13]. To address this challenge, the research community has progressively advanced the development of multimodal models, with the fusion of visual, signal, and textual information emerging as a particularly promising direction. These models demonstrate the capacity to simultaneously process and reason over heterogeneous data streams, unlocking new possibilities for tackling increasingly complex and diverse applications.

Notable progress in this domain includes the work of Liu et al., who extended instruction-based learning into the language–vision domain through the introduction of the LLaVA (large language and vision assistant) series, an end-to-end trained family of multimodal models [14]. By integrating a vision encoder with a large language model, LLaVA enables unified understanding across modalities and significantly enhances zero-shot generalization capabilities for novel tasks. Building on this foundation, LLaVA 1.5 further validated the effectiveness of employing a multilayer perceptron (MLP) as the vision–language connector [15]. This innovative approach encodes images through grid partitioning, allowing the model to handle arbitrary resolutions with flexibility and scalability.

To further improve the efficiency and adaptability of large language model fine-tuning, Luo et al. proposed the incorporation of lightweight connector modules between image encoders and large language models. This design not only facilitates seamless joint optimization but also integrates a routing mechanism that allows the model to automatically switch between single-modal and multimodal instruction processing. Leveraging this framework, the authors introduced LaVIN (large vision and instruction network), a novel multimodal model [16]. The LaVIN framework is underpinned by modality-mixed adaptation (MMA) and multimodal training strategies, enabling rapid adaptation to vision–language tasks without the need for extensive pretraining.

Extending the concept of multimodal fusion beyond two modalities, Yin et al. developed a multimodal instruction-tuning dataset that encompasses both images and point cloud data. This dataset emphasizes fine-grained detail and factual knowledge, with a thorough exposition of its construction methodology. In addition, they proposed a language-assisted multimodal instruction-tuning framework designed to optimize modality extension, accompanied by baseline models, experimental results, and in-depth analysis. Further expanding on these efforts, Zhang et al. introduced the large language and vision assistant with reasoning (LLaVAR) model [17]. LLaVAR aims to enhance multimodal understanding by leveraging rich text–image datasets and improves generalization to unseen tasks through instruction tuning, demonstrating strong performance in cross-modal reasoning tasks.

Summary of related work in multimodal vision-language models is shown in Table 1. Despite these advancements, current research remains predominantly focused on general-purpose vision-language applications, leaving a notable gap in the field of industrial intelligent perception and decision-making [16]. Existing multimodal large models are primarily trained on natural images and open-domain text, with a conspicuous absence of domain-specific industrial data such as manufacturing process documentation, equipment operation logs, and real-time sensor signals. This deficiency significantly restricts their utility in industrial environments characterized by high data heterogeneity and complex decision-making requirements. Consequently, the integration of existing multimodal large models with industrial intelligence demands, toward the development of an efficient, adaptable, and transferable industrial multimodal intelligent perception and decision-making system, remains an urgent and strategically important research frontier. Addressing these challenges will be critical to advancing intelligent manufacturing, predictive maintenance, and industrial safety management, and should constitute a priority for future research endeavors.

### 2.2. Current Applications of Large Language Models in Industrial Domains

In recent years, large language models have made transformative strides in natural language processing, computer vision, and multimodal learning. Despite these advancements, their application in industrial domains remains at an early, exploratory stage. Although select enterprises and research institutions have begun to integrate large language models into industrial intelligence pipelines, most existing industrial intelligence systems continue to rely heavily on traditional machine learning methods or rule-based decision-making frameworks. As a result, the full potential of large language models, particularly in cross-modal data fusion, intelligent perception, and autonomous decision-making, has yet to be fully leveraged. At present, applications of large language models in industrial contexts are primarily concentrated in intelligent manufacturing, predictive maintenance, intelligent quality inspection, and fault diagnosis. To better understand the current landscape and future development trajectories, this section examines two representative application scenarios: industrial vibration signal analysis and industrial anomaly detection.

In the domain of industrial vibration signal analysis, Wang et al. proposed a large-model-based analytical framework [18] that leverages a pretrained large-scale time series–text joint model to enhance the representational capacity of multidimensional signal features. This advancement has led to marked improvements in the diagnostic accuracy of bearing faults. Similarly, Ye et al. developed a large-model-based feature learning approach for vibration signals [19], employing a cross-modal knowledge distillation mechanism that enables the model to automatically extract key fault patterns from gearbox vibration data, thereby improving diagnostic precision and operational efficiency. Ribeiro et al. introduced a large language model trained on multichannel vibration signals that integrate visual, temporal, and textual information [20]. By employing cross-modal attention mechanisms and contrastive learning strategies, the model is capable of detecting and diagnosing six distinct types of motor faults. The vibration data are captured using accelerometers placed along two perpendicular axes, with the multi-head self-attention module independently processing inputs from different sensors to achieve efficient and robust feature extraction. Additionally, Li et al. proposed a self-supervised learning framework based on large language models [21] designed to diagnose gear pitting faults in scenarios where only limited raw vibration data are available.

In the field of industrial anomaly detection, multimodal large language models have demonstrated considerable potential in interpreting complex textual inputs and generating diverse outputs in combination with visual data. Jongheon et al. introduced WinCLIP [22], which encodes both textual descriptions and target images, while aggregating multi-scale visual features and text embeddings to ensure coherent alignment between modalities. Zhou et al. proposed AnomalyCLIP [23], a prompt-learning approach that learns generalized representations of normal and anomalous states, thereby enhancing the ability of the model to generalize across disparate domains. This technique reduces the dependency on manually crafted prompts and broadens the applicability of the model to industrial and medical anomaly detection scenarios. Building on this concept, Zhaopeng et al. introduced AnomalyGPT [24], a novel approach that simulates anomalies from normal samples to generate descriptive textual narratives of faults. A lightweight decoder based on visual–text feature matching was designed to directly compare local visual features with textual descriptions, enabling pixel-level anomaly localization. Through prompt learning, AnomalyGPT embeds industrial anomaly detection knowledge within multimodal large language models, creating prompt embeddings that allow for seamless integration of image data, anomaly localization outcomes, and user-provided textual inputs to facilitate robust anomaly detection and localization.

Summary of large model applications in industrial domains is shown in Table 2. While these advances in multimodal data fusion and industrial intelligent decision-making lay a solid foundation for further exploration, existing studies typically address narrowly defined tasks using specialized technologies. These approaches are typically optimized for unimodal data streams and lack the ability for deep cross-modal interactions, making them unsuitable for highly heterogeneous and complex decision-making environments in industrial settings. A review of the current literature reveals no comprehensive framework that systematically employs large-scale multimodal models for industrial data processing, deep cross-modal alignment, and intelligent decision optimization. Therefore, this research aims to establish an industrial multimodal intelligent perception and decision-making framework capable of concurrently processing diverse data sources, including visual imagery, sensor outputs, and production logs. Moreover, the framework will explore advanced methodologies for feature alignment, information fusion, and cross-modal reasoning mechanisms, enabling it to meet the demands of dynamic optimization tasks in complex and evolving industrial environments.

## 3. Materials and Methods

This chapter presents a multimodal methodology designed to unify visual data, production records, and textual descriptions for enhanced representation learning and cross-modal generation in industrial diagnostics and monitoring tasks. The proposed approach integrates heterogeneous data sources through an innovative semantic tokenization mechanism and a shared Transformer-based architecture, enabling more accurate and context-aware analysis of complex operational environments. By translating dense visual features, structured signal data, and sparse linguistic inputs into a common token space, this framework facilitates a more holistic understanding of machine behavior and degradation patterns.

### 3.1. End-to-End Pretraining Based on Semantic Tokens

**Figure 2:**
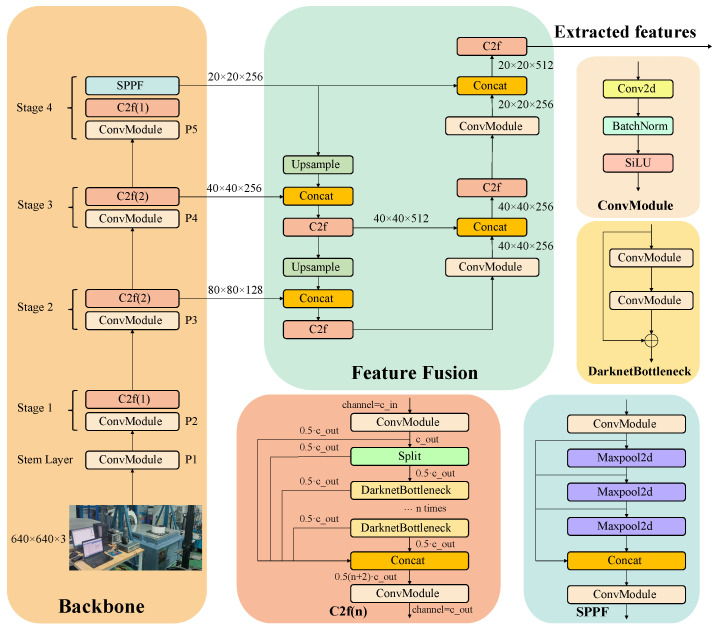
Comprehensive illustration of CNN-based visual encoder architecture.

To learn unified representations from three modalities: visual data, production records, and textual descriptions, this study proposed an end-to-end multimodal pretraining framework. First, the entire input image is processed using a trainable CNN-based visual encoder (CSPDarknet pretrained on ImageNet image dataset) [25], followed by a 3×3 convolution layer and a 4×4 max-pooling layer, which is shown in Figure 2. This encoding process generates grid-based feature maps without relying on predefined bounding boxes, ensuring that global contextual information critical for reasoning tasks is preserved and continuously updated during training. For the input image I, its feature V is obtained through the following formula:

(1) $$\begin{array}{c} V=E\left(I,\theta \right)\in R^{l\times c} \end{array}$$

where function E(·,θ) denotes a visual feature encoder parameterized by θ, producing a total of *l* feature vectors, each of dimensionality *c*.

To bridge the representational gap between dense visual features, structured production records, and sparse language tokens, a dynamic visual and signal dictionary was introduced. The dictionary clusters semantically similar visual features and indexed production data into shared semantic markers. These semantic embeddings are dynamically updated during training using momentum-based moving averages, and non-differentiability issues were resolved by leveraging a gradient stop technique. Specifically, the visual dictionary is defined as a matrix $D\in R^{k\times C}$ composed of *k* embedding vectors, each of dimensionality c. We denote the *j*-th vector in this dictionary as $d_{j}$. To associate a given visual feature $v_{i}$ with a corresponding dictionary entry, we identify its closest match in D by performing a nearest neighbor search:

(2) $$\begin{array}{c} h_{i}={argmin}_{j}{∥v_{i}-d_{j}∥}_{2} \end{array}$$

This lookup defines a mapping function f that assigns each input feature $v_{i}$ to its most semantically relevant embedding in D:

(3) $$\begin{array}{c} f\left(v_{i}\right)=d_{h_{i}} \end{array}$$

where the visual feature is represented using a semantically similar embedding vector. Initially, the dictionary D is populated with random embeddings and subsequently refined through a momentum-based moving average update during training. The update rule is given by:

(4) $$\begin{array}{c} {\hat{d}}_{j}\leftarrow \gamma d_{j}+\left(1-\gamma \right)v_{i} \end{array}$$

where ${\hat{d}}_{j}$ represents the updated embedding vector associated with the selected index, and γ∈[0,1] is a momentum coefficient that controls the update rate.

**Figure 3:**
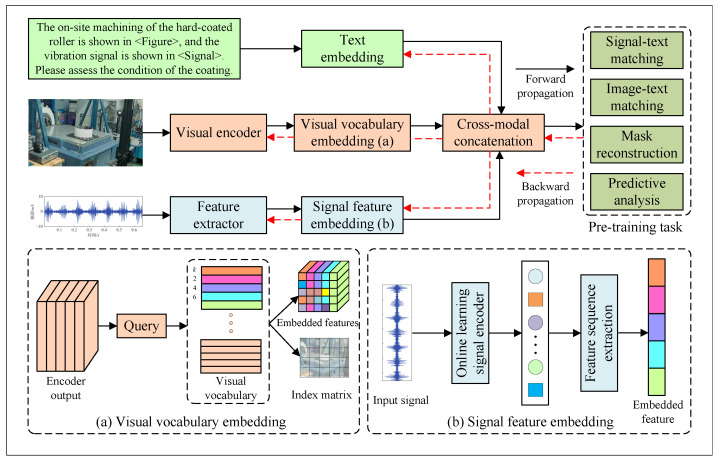
Workflow of generating dynamic visual and signal dictionary for bridging representational gaps.

Visual and production-related features are thus transformed into discrete semantic tokens comparable to text tokens, enabling joint processing. The workflow of generating a dictionary is shown in Figure 3.

The fused multimodal representations text embeddings from a WordPiece tokenizer, production record embeddings mapped into the same latent space, and dictionary-encoded visual features, concatenated and fed into a multi-layer Transformer network. The Transformer simultaneously handles modality fusion and task-specific decoding. Sinusoidal position encodings were applied to maintain spatial relationships in visual and production-record embeddings.

### 3.2. Unified Bidirectional Image–Text Generation Model

The model training objectives included three pretraining tasks: masked language modeling (MLM), masked visual modeling (MVM), and multimodal matching (MTM). For MLM, random text tokens were masked and predicted using surrounding text, visual, and production information. The goal of the pretraining task is to predict masked text tokens by maximizing their log-likelihood, conditioned on the surrounding context formed by both masked image tokens $X^{M^{\prime }}$ and masked text tokens $Y^{L^{\prime }}$, where $M^{\prime }$ and $L^{\prime }$ denote the masked positions. The Transformer model, parameterized by θ, and the objective minimizes the following cross-entropy loss:

(5) $$\begin{array}{c} L_{MLM}\left(\theta \right)=-\sum_{i=1}^{L^{\prime }}\log p_{\theta }\left(y_{i}|X^{M^{\prime }},Y^{L^{\prime }}\right) \end{array}$$

Since the layout structure remains fixed during this process, the objective encourages the model to capture meaningful relationships between the layout, the textual content, and the associated visual elements. For MVM, certain visual and production semantic tokens were masked and inferred from the context, with masking strategies adjusted to prevent trivial copying from neighboring regions. The loss function is defined as:

(6) $$\begin{array}{c} L_{MVM}\left(\theta \right)=-\sum_{m=1}^{M^{\prime }}\log p_{\theta }\left(x_{i}|X^{M^{\prime }},Y^{L^{\prime }}\right) \end{array}$$

MTM loss optimized the ability of the model to distinguish aligned from non-aligned triplets of images, production records, and text. The model takes contextual text and image as input and outputs a binary label of either “aligned” or “misaligned”, optimized using the binary cross-entropy loss:

(7) $$\begin{array}{c} L_{MTM}\left(\theta \right)=-\sum_{l=1}^{L-L^{\prime }}\log p_{\theta }\left(z_{l}|X^{M^{\prime }},Y^{L^{\prime }}\right) \end{array}$$

**Figure 4:**
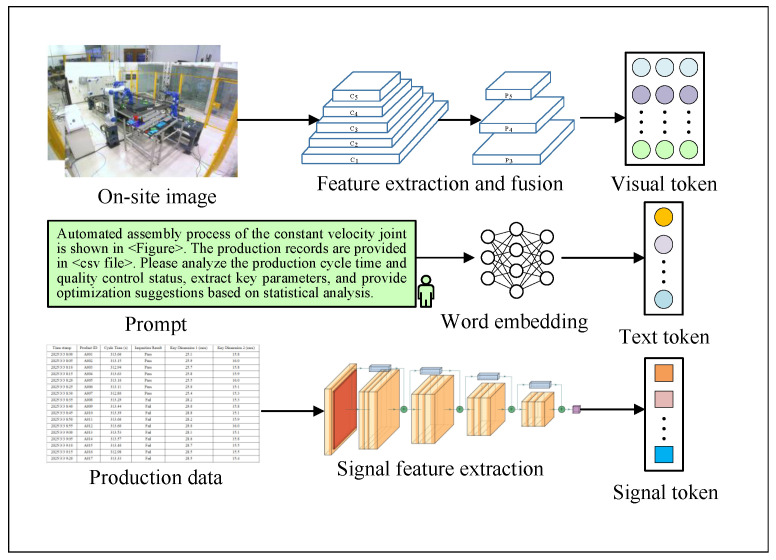
Aligning multiple modalities for input to the Transformer-based language model.

After the pretraining process, as shown in Figure 4, all modalities are unified and transformed into discrete token sequences that serve as input to the backbone large language model. Specifically, visual inputs are first processed by a CSPDarknet-based convolutional backbone [25] to extract high-level image features, which are subsequently quantized into visual tokens. Signal data are encoded via a one-dimensional convolutional ResNet architecture, capturing temporal patterns and structural information before being discretized into sequential tokens. Textual inputs are embedded using pretrained word embedding models, converting natural language into token representations compatible with the vocabulary space of the model. These unified token sequences from heterogeneous modalities are then fed into the large Transformer-based language model, enabling cross-modal representation learning and unified generation through a shared attention-based architecture.

**Figure 5:**
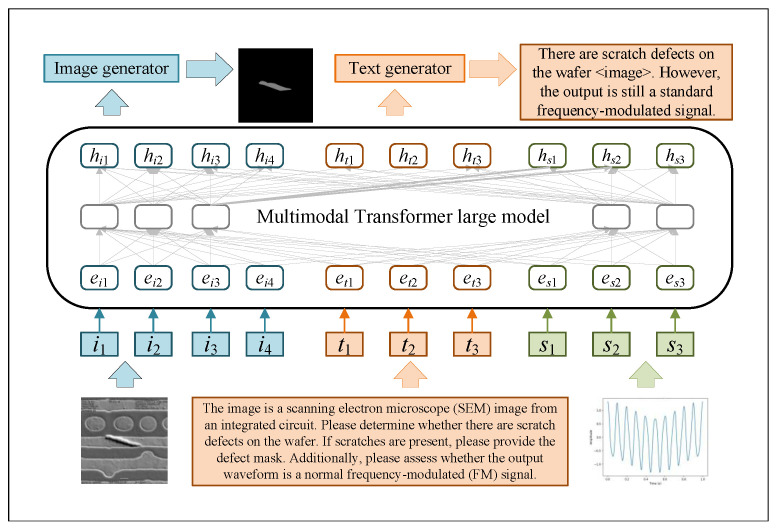
Unified Bidirectional Image–Text Generation Framework Based on Multimodal Transformer Architecture.

To enable the model to support multiple modal inputs and output text and images simultaneously, we propose a unified bidirectional image–text generation framework based on a multimodal Transformer architecture, which is shown in Figure 5. The model jointly handles image-to-text (I2T) and text-to-image (T2I) generation tasks within a single architecture, significantly reducing design complexity and improving parameter efficiency. The backbone of the framework is a multi-layer LLaMA-based Transformer consisting of multi-head self-attention, feed-forward layers, rotary positional embeddings, and normalization techniques. To stabilize training, this experiment follows CogView [26] by modifying the attention computation as follows:

(8) $$\begin{array}{c} \mathrm{softmax}\left(\frac{Q^{⊤}K}{\sqrt{d}}\right)=\mathrm{softmax}\left(\left(\frac{Q^{⊤}}{\alpha \sqrt{d}}K-\max\left(\frac{Q^{⊤}}{\alpha \sqrt{d}}K\right)\right)\times \alpha \right) \end{array}$$

where the hyperparameter α is set to 32. Most parameters are shared across I2T and T2I tasks, except for task-specific linear output layers. The Transformer accepts input sequences composed of visual and textual tokens and maps them into contextual embeddings for prediction. For T2I tasks, an additional image generator is employed to transform predicted 8×8 visual token grids into high-resolution 640×640 images.

Training is performed in two stages. First, token-level training using teacher-forcing optimizes cross-entropy loss for both I2T and T2I tasks. Second, sequence-level training addresses exposure bias. For I2T, self-critical sequence training (SCST) is applied with CIDEr-D rewards. For T2I, CLIP-based [27] image-level loss is introduced to promote semantic consistency between the generated images and the input text. This loss is computed based on the cosine similarity between the CLIP-derived embeddings of the generated image and the corresponding textual description, ensuring that both modalities are aligned within a shared semantic space. The T2I generation utilizes a mask-predict non-autoregressive decoding strategy to enhance inference speed, requiring only four sampling steps.

## 4. Results

This section presents a comprehensive suite of ablation studies designed to rigorously assess the performance of the proposed methodology. The ensuing analysis offers critical insights into the effectiveness of each component. Furthermore, comparative evaluations are included to benchmark the proposed approach against existing state-of-the-art methods. To carry out the experiment, the training process of the proposed and comparative models is performed on a GPU workstation, configured with 128.0 GB of RAM, an Intel Xeon E5-2698 CPU running at 3.6 GHz, and 8× NVIDIA Tesla V100 GPU. All algorithms are implemented in Python 3.10 using the PyTorch framework.

### 4.1. Results of Vision-Language Pretraining Tasks

This experiment uses the MS COCO [28] and Visual Genome (VG) datasets [29] for pretraining, as most vision-language pretraining tasks are built upon these datasets. To ensure fair evaluation and prevent data leakage, only the training and validation splits of these datasets are utilized during model training. The effectiveness of the model is assessed through two downstream tasks: image–text retrieval, evaluated on the Flickr30K dataset [30], and visual question answering (VQA), evaluated using the VQA 2.0 dataset [31].

#### 4.1.1. Image–Text Retrieval

Image–text retrieval comprises two different tasks: text retrieval (TR), where the goal is to find the most relevant textual description for a given image, and image retrieval (IR), which selects the most relevant image from a set of candidate descriptions. As a core task in vision-language learning, image–text retrieval underpins numerous real-world applications.

In line with common approaches, this experiment constructs mini-batches by sampling t image–text pairs with correct (aligned) annotations. For each image, the remaining t−1 texts in the batch are treated as negative (misaligned) samples. The retrieval task is formulated as a binary classification problem, where the model learns to distinguish between aligned and misaligned pairs. To make this prediction, the joint embedding derived from the Transformer’s output tokens is used. Because the image–text retrieval objective closely mirrors the image–text matching (ITM) task used during pretraining, the pretrained parameters naturally transfer well during fine-tuning.

The RAdam optimizer is used, with a learning rate of $10^{-4}$, decay rates of the moment estimates set to $\beta_{1}=0.9$ and $\beta_{2}=0.999$, and batch size set to 32. The model is trained for 30 epochs until convergence, with the learning rate halved empirically at the 5th, 10th, and 20th epochs.

VSE++ [32], SCAN [33], Unicoder-VL [34], BLIP-2 [35], $X^{2}$-VLM [36] and UNITER [37] algorithms are used for comparative evaluation. The experiments are conducted on the MS COCO [28] and Flickr30k [30] datasets, with the corresponding results presented in Table 3 and Table 4. The results demonstrate that the proposed pretraining approach outperforms previous vision-language pretraining methods on most metrics for both MS COCO and Flickr30k. The performance gains highlight the effectiveness of the approach in learning high-quality image–text embeddings through an end-to-end training framework, while also demonstrating the value of the visual dictionary in capturing semantically rich visual features.

#### 4.1.2. Visual Question Answering

Visual question answering (VQA) challenges a model to generate answers based on both an image and a corresponding natural language question. This task closely approaches intelligent AI, requiring the machine to perform cross-modal reasoning between vision and language in a human-like manner. In this experiment, VQA is modeled as a classification problem, where a multi-layer perceptron is trained to predict categorical answers. Binary cross-entropy loss is used as the optimization objective, with the same optimizer configurations and initial learning rate carried over from the pretraining phase.

ViLBERT [38], VisualBERT [39], LXMERT [40], BEiT [41], and UNITER [37] algorithms are used for comparative evaluation, with results summarized in Table 5. Among these, LXMERT serves as the most directly comparable baseline, sharing both the backbone architecture and pretraining datasets with the proposed method. The proposed method outperforms LXMERT by 5.84% on the test-dev set and 5.57% on the test-std set. Notably, LXMERT also utilizes additional out-of-domain datasets during pretraining. Even under this disadvantaged experimental setup, the proposed method still surpasses UNITER. The strong performance of the proposed method on the VQA task demonstrates the advantages of end-to-end pretraining approaches for visual question answering.

### 4.2. Results of Multimodal Alignment Tasks

To evaluate the effectiveness of the proposed feature embedding and modality alignment, ablation experiments were conducted by training a total of four different models. First, Model 1 serves as the baseline, utilizing only textual and layout information and trained with a masked language modeling (MLM) objective. Model 2 builds upon this baseline by introducing visual information, where image patches are linearly projected and incorporated as image embeddings into the model architecture. Then, Models 3 and 4 progressively apply the MVM and MTM objectives, respectively, for further pretraining based on Model 2.

**Figure 6:**
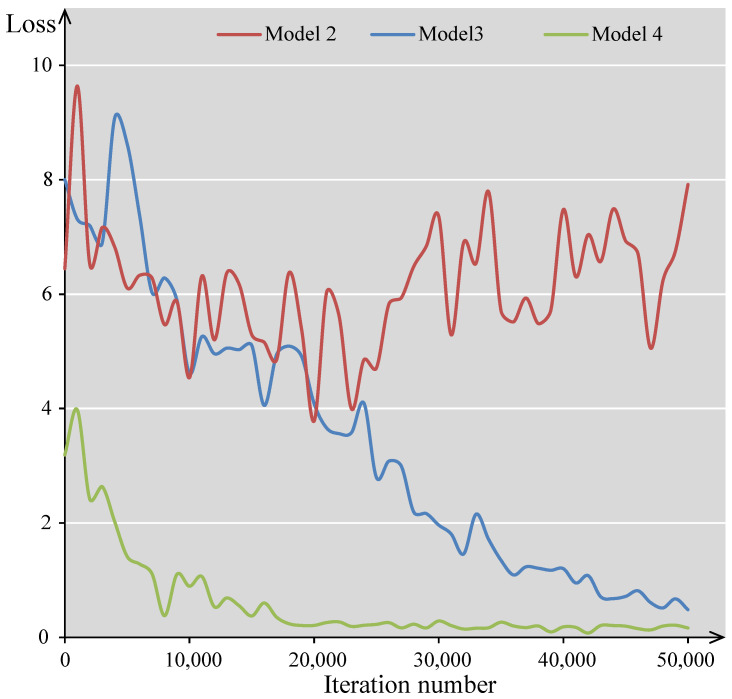
Loss convergence curves of the ablation models show that Model 2 fails to converge. By introducing the MVM objective, the loss begins to converge properly. The addition of the MTM objective further reduces the loss.

The variations in the loss functions during the fine-tuning process of the four models on multimodal datasets are illustrated in Figure 6. The experiments show that the loss function of Model 2 fails to converge. This may be due to the absence of supervisory signals in the image modality, resulting in ineffective alignment between visual and linguistic information. This cross-modal discrepancy likely leads to training instability, preventing the loss from converging.

For tasks that are primarily based on structured production data, such as the FUNSD [42] and CORD [43] datasets, and for image-centric tasks like RVL-CDIP [44] and PubLayNet [45], the performance metrics of different models are presented in Table 6. The results show that Model 1, despite lacking image embedding, achieves reasonably well on certain tasks. This highlights the significant contribution of the language modality, comprising both text and layout information, in document understanding. However, the overall performance remains suboptimal. Moreover, Model 1 is incapable of handling image-centric document analysis tasks, as these require visual modality input. Incorporating visual embeddings in Model 2 by simply appending linearly projected image patches to text embeddings leads to unexpected drawbacks. Specifically, it results in performance drops on the CORD and RVL-CDIP datasets and causes training instability (loss divergence) on PubLayNet. These outcomes indicate that, without a dedicated pretraining objective targeting visual modality, the model struggles to learn meaningful visual features. To address this, the masked visual modeling (MVM) objective is introduced, which randomly masks regions of the image input and requires the model to reconstruct them, thereby promoting better visual representation learning, preserving visual information all the way to the final layer of the model. A comparison between Model 3 and Model 2 demonstrates that MVM improves performance on both CORD and RVL-CDIP. Since the use of linear image embeddings alone already enhances performance on FUNSD, MVM does not provide further gains for that dataset. A comparison between Model 3 and Model 4 shows that the MTM objective leads to improvements across all tasks. Additionally, MTM reduces the loss of the visual task of PubLayNet. These findings demonstrate that MTM not only strengthens visual representation learning but also enhances the ability to capture cross-modal interactions.

### 4.3. Results of Bidirectional Image–Text Generation Model

This experiment evaluates the model on the MS COCO dataset, where each image is annotated with five image captions. The model is trained on the training set and evaluated on the validation set, with 25,000 images randomly selected for testing. For the image-to-text generation task, we follow the standard practice in most image captioning studies and evaluate our model on the Karpathy test split, a subset of the validation set containing 2500 images. The model is initialized from the pretrained X-LXMERT model [46] to enable direct comparison. This model adopts the LXMERT architecture [40] and is pretrained on the MS COCO Captions [28], Visual Genome [29], and VQA [47] datasets.

**Figure 7:**
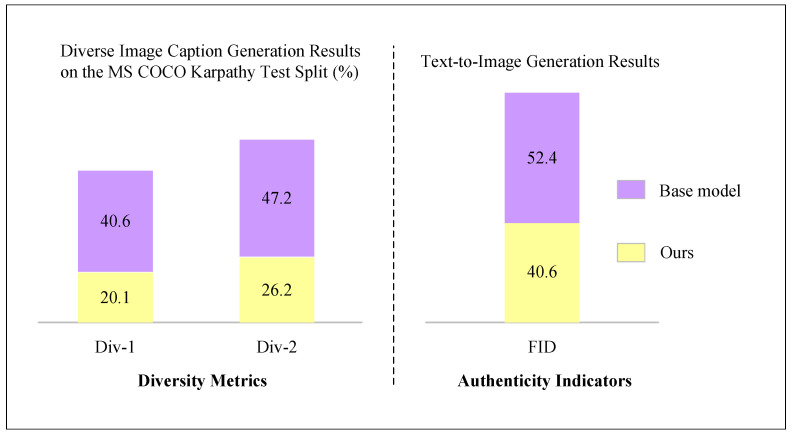
Comparison of the performance of models in diverse image caption generation and content-rich image generation.

To better assess the quality of generated images, a human evaluation is conducted for comparison with existing works, and the results are visualized in Figure 4, Figure 5, Figure 6 and Figure 7. Specifically, we compare our model with two state-of-the-art publicly available models: the GAN-based DM-GAN [48] and the Transformer-based X-LXMERT [46], both of which represent strong baselines. We also include a variant of our model without CLIP loss (denoted as “No CLIP”) in the comparison. We randomly sample 300 image captions from the MS COCO test set and use each model to generate images based on those captions. During evaluation, image–caption pairs generated by our model and the baselines are presented in random order to ten English-proficient volunteers with over ten years of learning experience. The volunteers are asked to judge which image (1) looks more realistic and (2) is semantically better matched to the original image caption.

To evaluate the realism of the generated images, we adopt the Fréchet inception distance (FID) [49], where a lower score indicates a closer match between the distribution of generated images and that of real images. For the generated image captions, we use n-gram diversity (Div-n) [50] to measure diversity, and CIDEr-D [51] to assess accuracy. This experiment is conducted on the Karpathy split [52] of the MS COCO Captions dataset [28], where each image is annotated with five captions, aligning well with the objective of our method. As illustrated in Figure 7 and Table 7, the proposed training strategy proves effective in producing diverse image captions. The caption sets generated by our method show significant improvements in diversity, with absolute gains of 51.7% in Div-1 and 74.7% in Div-2 compared to baseline methods. Although the captions generated by our method are more diverse and describe the image from different perspectives, they are slightly less accurate in terms of CIDEr-D compared to those generated by baseline models.

For the text-to-image generation task, incorporating diverse image descriptions improves the FID score, reducing it from 52.4 to 40.6. These results quantitatively confirm the capability of the system in producing effective diverse captions and content-rich image generation. This subsection proposes a bidirectional image–text framework capable of generating both multiple diverse image captions and semantically rich images. Built on a Transformer-based unified network, the approach considers the relationships among multiple input captions, effectively enhancing the diversity of generated captions. The effectiveness of the model is validated both quantitatively and qualitatively on the MS COCO Captions dataset.

## 5. Discussion

The proposed multimodal large language model-based intelligent perception and decision-making framework demonstrates substantial advancement in addressing the complex challenges associated with heterogeneous industrial data fusion. By effectively integrating visual information, production signals, and textual descriptions, our approach significantly enhances the capabilities of intelligent systems in smart manufacturing contexts. Through comprehensive evaluations, we have validated the robust performance of the model across diverse tasks including image–text retrieval, visual question answering, and multimodal alignment, thereby affirming its practical applicability and theoretical contribution to industrial automation.

Our experimental results indicate that the end-to-end pretraining strategy, complemented by semantic tokenization and unified bidirectional generation, effectively addresses the inherent challenges of modality disparity and representational gaps. Notably, our model consistently outperformed baseline and state-of-the-art methods across key evaluation metrics. In image–text retrieval tasks, our model exhibited superior precision, highlighting the effectiveness of our multimodal matching loss and masked modality prediction strategies in learning rich, discriminative embeddings. Similarly, substantial performance gains were observed in visual question answering tasks, demonstrating enhanced cross-modal reasoning capabilities facilitated by our proposed multimodal alignment framework.

The ablation studies provided valuable insights into the contributions of individual model components. The introduction of masked visual modeling was critical in stabilizing training and enabling effective visual information integration. Moreover, the multimodal matching objective further refined the ability of the model to align and exploit multimodal information, leading to noticeable improvements in downstream task performance. This layered approach not only confirms the importance of each pretraining task but also underscores the necessity of a structured and incremental training regime for optimal performance.

The unified bidirectional image–text generation framework introduced in this study significantly advances multimodal generation capabilities within industrial settings. By employing a shared Transformer architecture, our model successfully achieves seamless integration of image-to-text and text-to-image generation processes. The innovative use of a semantic dictionary for dynamic embedding updates further enables the model to bridge the semantic gap between modalities effectively, fostering a deeper and more nuanced understanding of complex industrial scenarios. Additionally, the incorporation of CLIP-based loss for text-to-image tasks substantially enhanced semantic consistency between generated images and their textual descriptions, indicating a promising direction for future multimodal generative models.

Despite the considerable advancements achieved, our study also highlights several limitations and areas for future research. First, the reliance on pre-existing datasets such as MS COCO and Visual Genome may limit its generalizability to highly specialized industrial datasets, which often exhibit unique and nuanced characteristics. Thus, developing dedicated large-scale industrial multimodal datasets could significantly enhance future model robustness and adaptability. Additionally, while our current approach emphasizes feature alignment and multimodal fusion, exploring advanced model interpretability techniques could further elucidate how multimodal decisions are made, enhancing user trust and facilitating practical deployment.

Finally, future research should explore extending the capabilities of models to incorporate real-time data streaming and dynamic adaptability within actual industrial production environments. Addressing scalability, real-time inference, and efficient incremental learning will be essential to ensuring the readiness of the framework for widespread industrial adoption. By continuously refining these aspects, the proposed framework has the potential to significantly transform intelligent manufacturing, enabling more autonomous, efficient, and adaptive industrial processes.

## 6. Conclusions

This study presents a comprehensive multimodal intelligent perception and decision-making framework specifically designed for smart manufacturing applications. By integrating visual imagery, production signals, and textual data into a unified representation learning structure, our proposed model substantially improves the accuracy and effectiveness of industrial decision-making processes. The empirical evaluation clearly demonstrates the superior performance of our approach over existing methodologies, highlighting the efficacy of our semantic tokenization mechanism, modality alignment strategies, and unified bidirectional generation framework.

The insights gained from this research not only provide a robust theoretical foundation for multimodal fusion but also establish practical guidelines for the deployment of intelligent industrial systems. Nonetheless, further exploration into dedicated industrial datasets, model interpretability, real-time adaptability, and scalable implementation remains crucial. Advancing these frontiers will undoubtedly enhance the applicability and resilience of multimodal intelligent systems, fostering significant progress toward more autonomous, efficient, and sophisticated smart manufacturing environments.

## Figures and Tables

**Table 1:** Summary of related work in multimodal vision-language models.

References	Authors	Problems Addressed	Solving Methods
[14]	Liu et al.	Bridging vision and language domains with unified multimodal understanding	Introduced LLaVA: Integrated a vision encoder with a large language model for end-to-end training
[15]	Liu et al.	Improving vision-language connectivity	Enhanced LLaVA with an MLP-based vision-language connector and grid-based image encoding
[16]	Liu et al.	Efficient and adaptive fine-tuning of large multimodal models	Proposed LaViN with lightweight connectors and modality-aware routing for joint optimization
[17]	Zhang et al.	Enhancing multimodal reasoning capability	Introduced LLaVAR with instruction tuning and reasoning-enhanced datasets

**Table 2:** Summary of large model applications in industrial domains.

References	Authors	Problems Addressed	Solving Methods
[18]	Wang et al.	Low representational capacity of vibration signals	Pretrained time series–text joint large model
[19]	Ye et al.	Difficulty in capturing gearbox fault patterns	Cross-modal knowledge distillation for feature learning
[20]	Ribeiro et al.	Lack of effective multi-fault motor diagnosis using multichannel signals	Multimodal model with cross-modal attention and contrastive learning
[21]	Li et al.	Lack of labeled data for gear pitting fault detection	Self-supervised learning framework for large fault detection models
[22]	Jongheon et al.	Cross-modal alignment challenges in anomaly detection	WinCLIP: multi-scale visual/textual encoding with prompt learning
[23]	Zhou et al.	Manual prompt dependency issues in anomaly detection models	AnomalyCLIP: generalized representations using prompt learning for anomaly detection
[24]	Zhaopeng et al.	Localizing and describing anomalies without labeled samples	AnomalyGPT: simulates anomalies and utilizes visual–text matching decoder for anomaly description

**Table 3:** Performance evaluation of the model on the image–text retrieval task using the MS COCO dataset. The results in bold indicate the best performance among the compared algorithms for each test set.

Algorithm	TR (1k Test Set)	IR (1k Test Set)	TR (5k Test Set)	IR (5k Test Set)
VSE++	87.3	81.4	68.6	57.1
SCAN	90.1	84.1	70.9	59.7
Unicoder-VL	92.4	86.4	72.3	61.0
BLIP-2	92.7	85.2	71.8	60.4
$X^{2}$-VLM	91.3	85.0	70.7	59.9
UNITER	93.1	87.2	73.5	62.1
Ours	**95.0**	**89.1**	**75.3**	**3.7**

**Table 4:** Performance evaluation of the model on the image–text retrieval task using the Flickr30k dataset. The results in bold indicate the best performance among the compared algorithms for each test set.

Algorithm	TR (R@1)	TR (R@5)	TR (R@10)	IR (R@1)	IR (R@5)	IR (R@10)
VSE++	57.0	64.1	64.9	47.1	59.8	63.2
SCAN	70.0	77.8	78.6	57.2	73.8	76.4
Unicoder-VL	86.1	97.4	98.9	71.8	92.5	95.4
BLIP-2	85.2	89.6	95.7	68.5	89.2	92.4
$X^{2}$-VLM	84.7	90.1	93.2	67.1	88.1	90.5
UNITER	85.4	96.2	97.1	70.5	88.9	93.8
Ours	**87.1**	**97.2**	**99.2**	**71.9**	**92.3**	**96.2**

**Table 5:** Performance evaluation of the model on the visual question answering task using the VQA 2.0 dataset. The results in bold indicate the best performance among the compared algorithms for each test set.

Algorithm	Test-Dev	Test-Std	FLOPs (GFLOPs)	Time to First Token (ms)
ViLBERT	63.05	63.62	60	415.4
VisualBERT	64.87	65.14	44	354.1
LXMERT	69.02	69.45	90	371.0
BEiT	68.10	68.87	155	584.2
UNITER	71.13	71.78	62	265.7
Ours	**73.05**	**73.32**	**38**	**204.3**

**Table 6:** Performance metrics of multimodal alignment tasks. The results in bold indicate the best performance among the compared algorithms for each test set.

Model	Image Embedding	Pretraining Objectives	FUNSD F1	CORD F1	RVL-CDIP Acc	PubLayNet mAP
1	None	MLM	79.39	85.44	75.73	N/A
2	Linear	MLM	84.07	90.52	94.46	N/A
3	Linear	MLM+MVM	86.75	94.90	93.09	92.38
4	Linear	MLM+MVM+MTM	**88.71**	**95.57**	**95.38**	**93.77**

**Table 7:** Diverse image caption generation results of the model on the MS COCO Karpathy test split. The results in bold indicate the best performance among the compared algorithms for each test set.

Method	Div-1 Diversity Metrics (%)	Div-2 Diversity Metrics (%)	CIDERr-D Acc (%)
Base model	20.1	26.2	**90.4**
Ours	**40.6**	**47.2**	78.4

## Data Availability

The datasets used in this study are openly available. The MS COCO dataset [28] and the Visual Genome dataset [29] used for pretraining tasks can be accessed through their respective official repositories. Additionally, the Flickr30K dataset [30] and VQA2.0 dataset [31] utilized for evaluation purposes are also publicly available. All supporting data can be obtained from the following sources: MS COCO: https://cocodataset.org/ (accessed on 1 May 2025). Visual Genome: https://homes.cs.washington.edu/~ranjay/visualgenome/api.html (accessed on 1 May 2025). Flickr30K: https://www.kaggle.com/datasets/hsankesara/flickr-image-dataset/data (accessed on 1 May 2025) VQA2.0: https://visualqa.org/ (accessed on 1 May 2025).
